# Assessing Strategy and Equity in the Elimination of Malaria

**DOI:** 10.1371/journal.pmed.1000312

**Published:** 2010-08-03

**Authors:** Naman K. Shah

**Affiliations:** 1 School of Medicine, University of North Carolina at Chapel Hill, Chapel Hill, North Carolina, United States of America; 2 School of Public Health, University of North Carolina at Chapel Hill, Chapel Hill, North Carolina, United States of America

## Abstract

Naman Shah critiques the malaria elimination agenda, arguing that it may only be feasible and equitable in limited settings.

**Summary Points**

- Recent dialogue around malaria elimination is laden with implicit assumptions.
- While the elimination of malaria may be both feasible and equitable in a few areas, globally the control tools that successfully reduce malaria burden may not be sufficient to interrupt transmission over long periods of time.
- A malaria elimination strategy may inadvertently increase inequity.

## Introduction

After the 2007 Bill & Melinda Gates Foundation meeting at which malaria eradication was declared back on the table [1], the charity was joined in its call by the World Health Organization (WHO) director general, the United States National Institutes of Health, and the Clinton Foundation, among others. A Gates-funded Malaria Elimination Group (MEG) has been convened [2], health ministers have delivered rousing speeches advocating elimination efforts, and scientists and policymakers have published eradication agendas and ideas. The impact of sustained advocacy is evident in the Global Malaria Action Plan that was commissioned by Roll Back Malaria and written by the Boston Consulting Group. The plan outlines a US$110.5 billion strategy for malaria control, including elimination and long-term eradication [3].

Not everyone is enthusiastic about these trends. Eradication skepticism has been cast by other groups, especially malariologists who remember the large-scale programs of the 1950s–1970s [4]. These campaigns achieved major successes, including local elimination, in a few areas but failed in many others. The practical differences between elimination and control have been highlighted by Lines et al., including the relative priority given to high and low burden areas, the choice and timing of interventions, and the degree of integration required with general health services [5]. Gosling and Chandramohan counter that the risks of promoting elimination are not unique. Under a strategy of sustained control, the use of similar approaches presents comparable risks regarding resistance to drugs and insecticides, management challenges, and the possibility of resurgence [6].

At the least, everyone can agree eradication is a powerful concept. It conveys a seductive sense of clear goals, time-bound effort, and scientific finality. This contrasts with caveats that are often obscured in fine print: that malaria eradication will be a several-decade effort, is contingent on the development of new technologies, and requires overall health system improvement. Consequently, promotion of the terms eradication and elimination has caused much confusion. Increased awareness about malaria is welcome, but grand plans also deserve deep scrutiny. Past successes of similar interventions and the comparability of tactics between control and elimination plans suggest that non-technological questions require our focus. Financing and political will are both vital and do receive some mention in today's rhetoric. What remain unspoken are issues that underlie much of the elimination debate: our understanding of the development of health and our understanding of equity.

## Is Malaria Elimination Limited by Control Technology?

Literature of late would lead one to infer that success in malaria elimination will be determined by the application of current interventions and the development of novel ones. According to the MEG's malaria elimination prospectus, more than 30 countries are either planning to or attempting to eliminate malaria [7]. The plan suggests that high population coverage of control tools (artemisinin combination therapies, rapid diagnostic tests, indoor residual spraying, and insecticide-treated bed nets) can eliminate malaria in these countries. For other areas, namely high-transmission zones, new control methods are deemed necessary in order to eliminate malaria. Malaria interventions are vital to reducing the economic and health burden of the disease. But can the application of biomedical tools explain long-term changes in malaria incidence?

Historically, there are many examples of decreases in malaria and all-cause burden where interventions were made widely available. However, fewer cases do not necessarily lead to zero transmission. An equal number of cautionary tales highlight the risk of malaria resurgence due to technical, financial, and political disruptions in control programs. Matters of “human ecology”, in no small part, contributed to failures in previous malaria eradication efforts [8]. We must be clear about what leads to sustained reductions in malaria incidence if we hope to avoid repeating the past. The primary determinant of malaria transmission is the vector and its ability to interact with people. Vector ecology and human contact are in turn dictated by physical and social settings. Thus, we can deduce that differences in environmental features can explain the variation in malaria incidence between populations.

Among components of the physical environment, temperature, rainfall, and humidity are less amenable to deliberate alteration. However, factors such as urbanization, employment, housing quality, and industry, which follow economic development, are largely products of public policy. Communities with ready access to schools, clinics, and markets are healthier and they will remain so in the absence of any intervention aimed at them or the mosquitoes around them. Forty years ago, Thomas McKeown arrived at similar conclusions regarding long-term changes in mortality [9]. While there are reasonable critiques of McKeown's methods, his provocative question on the value of clinical interventions focused at the individual or community versus broader efforts to alter forces that distribute resources affecting population health remains relevant.

Indeed, numerous case studies point to general development as a key determinant of long-term changes in malaria incidence. Malaria epidemics in India due to the “tropical aggregation of labor”, coupled with exploitative conditions of employment, were documented in tea estates by Sir Rickard Christophers as early as 1907 [10]. The post-independence expansion of dams and irrigation in former colonies altered malaria risk in settings worldwide [11]. Continuing the agricultural theme, medical historian Margaret Humphreys concluded that malaria elimination in the southeastern United States was driven by farming policies that removed poor sharecroppers from mosquito-infested swamplands rather than by efforts to spray DDT or build drainage ditches [12]. A more contemporary analysis in Vietnam from 1980 to 2000 attributes the improved malaria scenario to a confluence of changes from improved living standards, less work migration, more health workers, and greater political stability [13]. New drugs and insecticide-treated nets also contributed to control in Vietnam, but by the time of their scale-up in the mid-1990s, malaria incidence was already on a precipitous decline [13]. Earlier this year, officials from Brazil judged malaria elimination impractical due to the difficulty in reducing the economic and social risk factors that determine its incidence there [14].

The contribution of malaria interventions to the reduction of disease burden is invaluable. Control tools also decrease long-term transmission by helping to enable necessary social changes, but anti-malarial measures alone are unlikely to achieve elimination in most places. Engaging in historical reflection is important so, as Krieger and Birn argue, “…we may resist the hubristic belief that, as public health professionals, we have all the answers or can by ourselves improve the public's health without efforts to ensure social and economic justice” [15].

## Will a Malaria Elimination Strategy Advance Equity?

The brunt of malaria burden, as with many diseases, is borne by the most disadvantaged members of society [16]. Since the distribution of the burden is unfair, one might assume any anti-malaria activity improves equity—i.e., the benefits are equally distributed among members of a community. Empirical studies of who profits from the distribution of public goods (whether drugs or bed nets), however, suggest programs tend to favor those who are better off [17]. The 200-plus page MEG malaria elimination prospectus devotes a section to “equity impact” [7]. Unfortunately, the extent of analysis is a blanket claim that “…elimination programs will, by reaching remaining segments of the population, almost surely prove to be equity enhancing”. While the actual elimination of malaria would be equitable, elimination may fail, and meanwhile elimination programs may not distribute benefits more equitably than present efforts. Most gains of equity in the receipt of goods would result from successful universal coverage, which is already part of many control strategies and not unique to elimination. Overall, the appraisal is limited. Disease targeting alone will not ensure equity; a more complex consideration of equity in malaria elimination is needed.

First, elimination efforts may decrease equity between regions in terms of the allocation of resources proportional to disease burden. Current plans target richer countries and richer provinces within endemic countries [7]. Malarious zones surrounding malaria-free areas present a risk for the re-establishment of transmission. Several documents advocate an “attack at the margins” plan as the method by which to embark on global malaria elimination [3],[7]. Proponents believe that in order to eliminate malaria in the “heartland”, elimination in border countries would be prerequisite. They advocate for elimination efforts to be concentrated where malaria attack rates are low and unstable. Such areas are, by and large, more prosperous. In theory, increased inequity will be temporary until global eradication is achieved. However, the high level of uncertainty about when or even if eradication could occur suggests it may be inappropriate to trade equity in the present for anticipated equity in the future. Another counterargument is the assertion that targeting areas of low transmission is not exclusive with achieving universal coverage in more malarious locales. The reality, though, is that available resources are inadequate or at least finite. Neither local nor international health funding is zero sum, but some level of opportunity cost undeniably exists. Challenges in how to allocate resources within a country and between countries are already present; an elimination focus alters the criteria used to make decisions. Malaria burden and poverty need not be the sole criteria, but attempting elimination at the expense of general control will produce short-lived victories.

Second, elimination efforts may decrease equity in terms of the allocation of resources proportional to disease priority in overall health. Improved malaria outcomes may not be synonymous with improvements in total health. Disease-specific programs can achieve major health gains and help improve the broader health system. Promoting a single agenda to the exclusion of other priorities, though, could squander those very gains. For example, the Global Malaria Action Plan stresses the need to maintain awareness of and support for malaria control [3]. This seems sensible. Unfortunately, that need is placed within the context of maintaining prominence over “competing global health and development priorities”. Such narrow communication and the attitude it embodies are unfortunate. For all of us committed to public health, the setting of priorities must be an exercise based in fairness. What constitutes fairness is a difficult question whose answer will vary broadly, even among members of the same community. At the least, it cannot be solely dictated by advocates. Similar conflicts may exist in practice as in advocacy. The impact a re-orientation towards elimination could have on generalized health systems is concerning [5]. In many countries, components of malaria control are delivered through the primary care system. Within an elimination scenario, malaria interventions may be prioritized and delivered at the expense of other health services routed through the same system.

Finally, elimination efforts may decrease equity in the planning process in terms of participation and influence proportional to the stake in malaria control. Global plans do stress that within-country and within-district allocation should be decided locally, albeit pending donor approval [3]. Money speaks; but where does the primary concern for malaria control lie? The answer is unequivocal: endemic countries, or better yet, endemic communities. Many public health workers instead only allude to the malaria control interests of some “global health community” [18]. We must recognize the peril of this attribution. Such language, even if unintentional, reflects a subtle appropriation of responsibility and devalues local decision makers. The exaggeration of a self-appointed mandate is easy to explain. The dearth of voice from program managers and others who understand malaria along dirt roads of the rural tropics creates a one-sided perspective. Which groups then have prominent voices, and where are they based? The answer is uncomfortable: air-conditioned towers in cities of the West. Ultimately, the degree of separation between those who plan policy from the reality of malaria does not inspire confidence. This can change. Country ownership already exists; country agency, at least where governments are dependent on international financing, requires partners to provide support without superseding [19]. Concerns about equity in the planning process are by no means exclusive to elimination. Similar conflicts exist in planning malaria control. Nonetheless, equity in decision-making may be more compromised under a malaria elimination scenario as the stakes become elevated.

## Conclusions

The elimination of malaria using control tools may be feasible and equitable in limited settings. However, these assumptions may not be valid globally. The potential cost of not addressing these concerns includes a great waste of effort, funds, and goodwill.
